# Spine alignment influences shoulder range of motion and scapular orientation: A systematic review from the FP‐UCBM Shoulder Study Group

**DOI:** 10.1002/jeo2.70604

**Published:** 2026-04-16

**Authors:** Pietro Gregori, Mauro La Bruna, Giuseppe Francesco Papalia, Giancarlo Giurazza, Clemente Caria, Michele Paciotti, Fabrizio Russo, Edoardo Franceschetti, Umile Giuseppe Longo, Rocco Papalia

**Affiliations:** ^1^ Fondazione Policlinico Universitario Campus Bio‐Medico Roma Italy; ^2^ Research Unit of Orthopaedic and Trauma Surgery, Department of Medicine and Surgery Università Campus Bio‐Medico di Roma Roma Italy

**Keywords:** kyphosis, scapulothoracic joint, shoulder biomechanics, shoulder ROM, spine morphology

## Abstract

**Purpose:**

The relationship between spine orientation and the shoulder active range of motion (ROM) has been an ever‐growing topic in the last few years. This systematic review aimed to assess the epidemiological association between spine morphology and shoulder function.

**Methods:**

The PRISMA (Preferred Reporting Items for Systematic Reviews and Meta‐Analyses) guidelines were followed. A literature search was performed on PubMed, Scopus, and Cochrane library. Randomised controlled trials (RCTs) and cross‐sectional studies, analysing the correlation between shoulder ROM and Cobb angle, kyphosis index, scapular tilt, forward scapular posture, functional scores in adults were selected. Finally, data of interest were extracted and analysed.

**Results:**

A total of five studies were included in this study. The study included a total number of 624 participants and 887 shoulders. The mean age 40 years old, ranging from 17.1 to 67. The most significant correlations (*p* < 0.0003) were that greater thoracic kyphosis was associated with increased scapular internal rotation (*R *= Left −0.081 Right = −0.065) (*p* < 0.0003), and that greater scapular anterior tilt was associated with increased scapular internal rotation (*R* = 0.29, *p* < 0.001). Higher thoracic kyphosis was correlated with reduced shoulder abduction (*R* = −0.163, *p* < 0.005) and flexion (*R* = −0.118, *p* < 0.05), whereas greater lumbar ROM was associated with increased abduction and flexion (*R* = 0.119, *p* < 0.05 and *R* = 0.166, *p* < 0.005). In addition, higher thoracic kyphosis was correlated with reduced shoulder adduction (*R* = −0.72, *p* < 0.05).

**Conclusions:**

Sagittal spine morphology shows frequent and significant correlations with shoulder active ROM. In particular, greater thoracic kyphosis is associated with increased scapular internal rotation and reduced shoulder abduction, adduction and flexion, while higher lumbar mobility correlates with improved shoulder elevation. These findings confirm the biomechanical interdependence between spinal alignment and shoulder kinematics.

**Level of Evidence:**

Level III.

**Abbreviations**
ADLS: activities of daily living
BMI: body mass index
CE: ceiling effects
FLEX‐SF: flexilevel scale of shoulder function
KI: Kyphosis Index
LOE: level of evidence
PRISMA: Preferred Reporting Items for Systematic Reviews and Meta‐analyses
PROMs: Patient‐Reported Outcomes Measures
QUADAS‐2: Quality Assessment of Diagnostic Accuracy Studies‐2
RCTs: randomised controlled trials
ROM: ange of motion
RTSA: reverse shoulder arthroplasty
VAS: Visual Analogue Scale

## INTRODUCTION

It is generally agreed that age‐related changes in the spine and shoulder cause kyphosis, spinal sagittal imbalance, and limited shoulder range of motion (ROM) [17]. Activities of daily living (ADLs) are significantly influenced by spinal disorders and restricted shoulder ROM. Preserving functional independence in elderly individuals is therefore essential, not only to maintain quality of life but also to reduce reliance on nursing care and to limit healthcare costs in an aging society.

Biomechanical studies show that a relationship between spine and shoulder ROM exists, with manipulation of the spine and ribs improving the symptoms of shoulder pain and impingement syndrome without rehabilitation [2, 26]. The role of the thoracic spine as a key component for the function of the upper limb has also been demonstrated as well as the shoulder playing a fundamental role in the motility of the thoracic and cervical spine [6]. Approximately 9°–15° of thoracic extension ROM seems to be required for a full bilateral shoulder flexion in both younger and older populations, however, these parameters may vary significantly due to the inherent variability across individuals and conditions [21]. Moreover, a significantly increase on active shoulder abduction ROM in an erect posture in relationship to a slouched posture has been shown recently [14]. Although several studies have suggested a link between thoracic kyphosis and shoulder disorders, Yamamoto et al. reported that altered posture independently predicts both symptomatic and asymptomatic rotator cuff tears, highlighting the role of spinal alignment in prevention strategies and in tailoring rehabilitation for shoulder pathology [28].

When focusing on the scapula, increased scapular protraction has shown potential compression distress under the acromion and subacromial tissues, including the subacromial bursa and rotator cuff [15]. There appears to be a link between increased scapular internal rotation or anterior inclination of the scapula and progressive thoracic kyphosis with lowering of the scapula relative to the thorax.

By relating these data into preoperative reverse shoulder arthroplasty (RTSA) planning, it seems clear that an examination of the patient′s posture could provide an estimate for choosing the optimal positioning for the humeral and glenoid component [23]. The objective of this study was to evaluate the association between spinal posture, particularly thoracic kyphosis, scapular orientation and shoulder ROM, with an analysis of the literature, with the aim of understanding their implications for function and surgical planning in reverse shoulder arthroplasty. The hypothesis was that spinal posture could affect shoulder mobility and the positioning of the scapula.

## MATERIALS AND METHODS

A literature search according to Preferred Reporting Items for Systematic Review and Meta‐Analyses (PRISMA) 2020 guidelines was performed by two reviewers working independently.

### Eligibility criteria

We included observational prospective or retrospective studies in English, which investigated a correlation between the coronal or sagittal axis of the spine, joint mobility and the ROM of the shoulder. We excluded studies that included the paediatric population, patients undergoing corrective spinal surgery or corrective rehabilitation. Moreover, we excluded studies that did not report a correlation index between spine and shoulder outcomes. The comparison analysis was based on the evaluation of clinical scores, shoulder ROM, anterior and posterior tilt of the scapula, kyphosis angle and grade of scoliosis, in order to estimate changes in shoulder ROM, strength or functional performance.

### Informational source and search

The systematic search was performed by the FP‐UCBM Shoulder Study Group on May 29, 2024. The complete PubMed search strategy was as follows: (“shoulder”[MeSH Terms] OR “shoulder”[All Fields] OR “shoulders”[All Fields] OR “glenohumeral joint”[All Fields]) AND (“kyphosis”[MeSH Terms] OR “thoracic kyphosis”[All Fields] OR “spinal curvature”[All Fields] OR “postural alignment”[All Fields]) AND (“posture”[MeSH Terms] OR “spinal posture”[All Fields] OR “sagittal balance”[All Fields]). No restrictions were applied regarding publication date, study design, or language. The search strategy was adapted for Scopus and the Cochrane Library using equivalent keywords and database‐specific syntax.

### Study selection

The first search was based on title and abstract of the articles selected with the reported string; subsequently, eligible articles were read in full text. Finally, a manual search of the reference list of the included articles to find additional manuscripts was performed and citation mapping was not performed.

### Data collection process

The following demographic characteristics have been extracted: authors, year of publication, type of study, level of evidence (LOE), number of participants, number of shoulders, age, gender, body mass index (BMI). Moreover, for the analysis of the results of the included studies, the correlations between scapular or shoulder ROM, such as abduction, flexion, external and internal rotation, and spinal parameters, such as thoracic kyphosis, thoracolumbar flexion, thoracolumbar extension, scoliosis, thoracic and lumbar ROM were assessed. For these correlations, the Pearson correlation coefficient and *p*‐value were reported.

Two reviewers independently extracted data from the eligible studies using a default data extraction shaped in accordance with the protocol. For each study, the criteria regarding the epidemiological characteristics of the participants (age, sex, number of shoulders evaluated) and the evaluation of the results (correlation between shoulder ROM and Cobb angle, scapular tilt, forward scapular posture and functional scores) were extracted. The objective was the analysis of the correlation coefficient and related statistical significance between shoulder ROM and alterations in the Cobb angle of the spine.

### Risk of bias assessment

The Quality Assessment of Diagnostic Accuracy Studies‐2 (QUADAS‐2) tool was used to assess the risk of bias of the included studies, examining four domains: patient selection, index test, reference standard and flow and timing.

## RESULTS

### Results of the search

**Figure 1:**
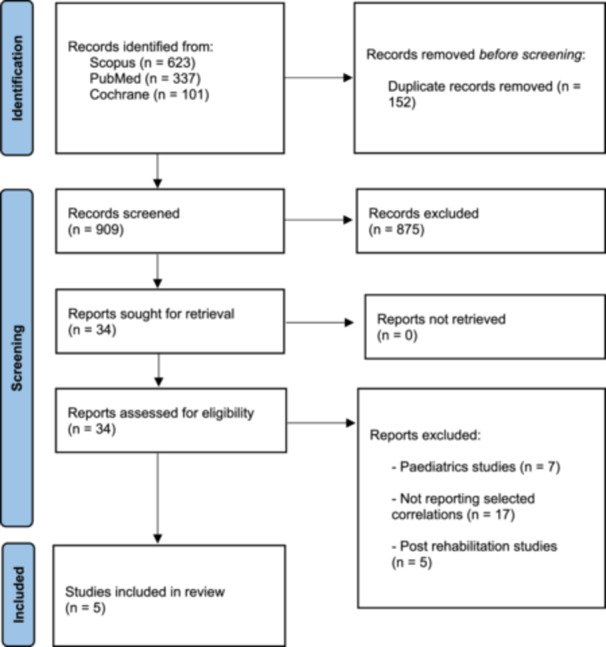
PRISMA 2020 flowchart.

All steps of the literature search, screening, eligibility assessment, and inclusion were conducted in line with PRISMA recommendations, and the complete process is illustrated in (Figure 1).

### Included studies

A total of five studies (three cross‐sectional and two prospective cohort studies) were included. These investigations analysed alterations in shoulder ROM and their statistical correlations in patients with sagittal or coronal spinal malalignment [12, 13, 16, 18, 23].

The quality assessment showed that all the included studies presented a low risk of bias in patient selection, index test, and flow and timing domains, whereas the reference standard exhibited an unclear risk of bias. bias as assessed by the QUADAS‐2 tool (Table 1).

**Table 1:** QUADAS‐2.

Study	Risk of Bias				Applicability concerns		
	D1	D2	D3	D4	D1	D2	D3
Heather et al. (2021)	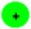	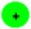	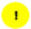	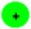	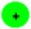	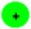	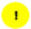
Moroder et al. (2020)	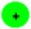	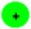	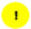	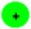	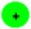	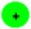	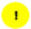
Imagama et al. (2014)	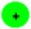	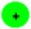	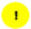	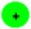	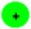	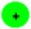	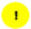
Lee JH et al. (2014)	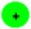	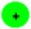	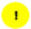	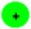	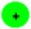	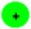	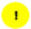
Lin JJ et al. (2010)	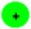	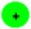	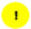	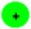	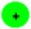	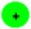	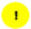

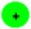
 Low risk

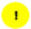
 Unclear risk

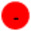
 High risk

D1 Patient Selection

D2 Index Test

D3 Reference Standard

D4 Flow and Timing

### Demographic data

The study included 624 participants, accounting for a total of 887 shoulders, with a mean age of 40 years (range, 17.1–67). Gender distribution in our total population was 60% female (369 patients) and 40% male (255 patients), ranged from 26% to 64% of women premidonancy for single study, while one study included only females (Table 2).

**Table 2:** Study details.

Author (year)	Type of study	LOE	No of patients	No of shoulder	Mean age (range)	Sex	Country
						F (%) M (%)	
Johnston and Drake (2021) [13]	Cross‐sectional study	II	163	326	21 (18–35)	104 (64%) 59 (36%)	Canada
Moroder et al. (2024) [24]	PCS	II	100	200	61.1	26 (26%) 74 (74%)	Germany
Imagama et al. (2014) [12]	PCS	II	317	317	67 (50–90)	203 (64%) 114 (36%)	Japan
Lee et al. (2015) [16]	Cross‐sectional study	II	18	18	33.8 ± 11.2	10 (55%) 8 (45%)	South Korea
Lin et al. (2010) [18]	Cross‐sectional study	II	26	26	17.1	26 (100%) 0 (0%)	Taiwan

*Note*: BMI was reported in two studies, the mean values were 21.19 and 24.2 kg/m^2^.

Abbreviations: LOE, level of evidence; PCS, prospective cohort studies.

### Correlation of clinical outcomes

Main clinical outcomes were reported in Table 3, while the extracted correlations are summarised in Table 4.

**Table 3:** Functional results at final follow‐up.

Study	Thoracic kyphosis (Cobb Angle)	Shoulder flexion	Shoulder abduction	Shoulder internal rotation	Scapular anterior tilt	Scapular upward rotation	Scapular protraction	Functional scores
Johnston and Drake [13]	9.2 ± n.r (KI)	r 169° ± n.r. l 169° ± n.r.	r 176° ± n.r l 175° ± n.r.	r 53° ± n.r. l 55° ± n.r.	r 71° ± n.r. l 69° ± n.r.	n.r.	n.r.	n.r.
Moroder et al. [24]	44° ± 11.2°	n.r.	n.r.	41.4° ± 5.2°	19.4° ± 8.6°	13.3° ± 5.1°	88.4° ± 4.7°	n.r.
Imagama et al. [12]	43.1°± n.r.	177° ± n.r.	175.3° ± n.r.		n.r.	n.r.	n.r.	3.7 ± n.r. (VAS pain)
Lee JH et al. [16]	33.77° ± 8.37°	n.r.	n.r.	54.08° ± 13.13°	n.r.	n.r.	n.r.	n.r.
Lin et al. [18]	34.0° ± 7.3°	n.r.	n.r.	n.r.	10.4° ± 4.6°	14.3° ± 6.2° 6.2° ± 6.4°	n.r.	35.5 ± 4.6 (FLEX‐SF)

*Note*: Values are reported as mean ± standard deviation (SD) unless otherwise specified.

Abbreviations: FLEX‐SF, flexilevel scale of shoulder function; KI, Kyphosis Index; l, left shoulder; n.r., not reported; r, right shoulder; VAS, Visual Analogue Scale.

**Table 4:** Main outcomes rates.

Author	Sample	Method used and parameters evaluated	Correlation	*p* value	*R*
Johnston and Drake [13]	163 asymptomatic, right hand dominant, young adults	Manual goniometric ROM; thoracic/lumbar motion with inclinometer in asymptomatic young adults.	Thoracic kyphosis and shoulder abduction	<0.0003	**L** 0.086 **R** 0.151
			Thoracolumbar flexion and shoulder abduction		**L** 0.211 **R** 0.238
			Thoracolumbar extension and shoulder abduction		**L** 0.066 **R** 0.084
			Thoracic kyphosis and shoulder flexion		**L** −0.118 **R‐**0.159
			Thoracolumbar flexion and shoulder flexion		**L** 0.159 **R** 0.221
			Thoracolumbar extension and shoulder flexion		**L** 0.077 **R** 0.077
			Thoracic kyphosis and scapular external rotation		**L** −0.028 **R** −0.014
			Thoracolumbar flexion and scapular external rotation		**L** 0.002 **R** −0.165
			Thoracolumbar extension and scapular external rotation		**L** 0.232 **R** 0.180
			Thoracic kyphosis and scapular internal rotation		**L** −0,081 **R** −0,065
			Thoracolumbar flexion and scapular internal rotation		**L** 0.079 **R** −0.039
			Thoracolumbar extension and scapular internal rotation		**L** 0.163 **R** −0.023
Moroder et al. [24]	200 shoulders in 100 patients	CT with 3D reconstruction; thoracic kyphosis and scapular orientation in adults.	Thoracic kyphosis and scapular internal rotation	<0.001	0.27
			Anterior tilt and scapular internal rotation	<0.001	0.29
			Scapular translation and scapular internal rotation	<0.001	0.57
Imagama et al. [12]	317 subjects (114 males and 203 females)	Motion analysis system with digital reconstruction for evaluation of thoracic/lumbar ROM and shoulder kinematics in elderly cohort.	Thoracic kyphosis and shoulder abduction	<0.005	−0.163
			Thoracic ROM and shoulder abduction	>0.05	0.035
			Lumbar ROM and shoulder abduction	<0.05	0.119
			Thoracic kyphosis and shoulder flexion	<0.05	−0.118
			Thoracic ROM and shoulder flexion	>0.05	−0.057
			Lumbar ROM and shoulder flexion	<0.005	0.166
Lee et al. [16]	18 subjects	Digital image analysis; thoracic kyphosis and scapular posture in young adults.	Thoracic kyphosis and shoulder adduction	<0.05	−0.72
			Thoracic kyphosis and forward scapular posture	0.077	−0.43
Lin et al. [18]	13 females with idiopathic scoliosis and 13 females without scoliosis	Motion analysis; scapular kinematics in adolescent females with/without scoliosis.	Idiopathic scoliosis and shoulder flexion	0.01	
			Idiopathic scoliosis and anterior tilt of the scapula	0.006	
			Scoliosis (convex side) and scapular anterior tilt	<0.05	
			Scoliosis (concave side) and scapular upward rotation	<0.05	

Abbreviations: CT, computed tomography; L, left shoulder; R, right shoulder; ROM, range of motion.

### Thoracic kyphosis

In the study by Imagama et al. and Johnston and Drake [12, 13] subjects with greater thoracic kyphosis showed reduced shoulder flexion.Imagama et al. further demonstrated that both increasing thoracic kyphosis angle and spinal inclination were important risk factors for decreased shoulder flexion, while greater thoracic kyphosis combined with weak back muscle strength was a significant risk factor for decreased shoulder abduction (*p* < 0.05). Overall, shoulder flexion was influenced by thoracic kyphosis, thoracic ROM, and lumbar ROM, whereas shoulder abduction decreased with increasing thoracic kyphosis. In contrast, greater thoracic and lumbar ROM were associated with increased shoulder flexion and abduction (Table 4).

Lee et al. [16] reported that a greater degree of forward scapular posture was associated with a larger thoracic spine angle. This alignment was also linked to slightly reduced glenohumeral horizontal adduction and moderately reduced glenohumeral internal rotation. In the study by Moroder et al. [23] scapular internal rotation was found to increase in association with thoracic kyphosis, scapular protraction, anterior tilt, and greater scapular translation (data were reported in Table 3).

### Thoracolumbar flexion and extension

The most significant findings in the study by Johnston and Drake [13] were that shoulder abduction and flexion increased with greater thoracolumbar flexion (R Left = 0.211; Right = 0.238; and R Left = 0.159; Right = 0.221, respectively), both highly significant (*p* < 0.0003). In addition, scapular external rotation increased with greater thoracolumbar extension (R Left = 0.232; Right = 0.180).

Other associations were weaker and less consistent. Shoulder abduction and flexion changed only minimally with thoracolumbar extension. Scapular internal rotation showed only slight changes with thoracolumbar flexion (R Left = 0.079; Right = –0.039) and extension (R Left = 0.163; Right = –0.023). Finally, scapular external rotation was not meaningfully affected by thoracic kyphosis or thoracolumbar flexion.

### Idiopathic scoliosis

In the study by Lin et al. [18], which investigated the relationship between idiopathic scoliosis and shoulder ROM, significant alterations in shoulder kinematics were reported. On the convex side, patients showed greater anterior tilt of the scapula in resting position (*p* = 0.006), whereas no significant differences were found in scapular upward rotation, posterior tipping, or scapulohumeral rhythm. On the concave side, patients demonstrated greater scapular upward rotation in resting position (*p* = 0.01).

## DISCUSSION

As previously hypothesised, this systematic showed an impact of spine morphology on scapular and shoulder articular mobility and anatomical orientation. The most significant correlations were observed between greater thoracic kyphosis and increased scapular internal rotation, as well as between greater scapular anterior tilt and greater scapular translation. Higher thoracic kyphosis was correlated with reduced shoulder abduction and flexion, whereas greater lumbar ROM was associated with increased abduction and flexion. In addition, higher thoracic kyphosis was correlated with reduced shoulder adduction. To our knowledge, this investigation represents the first systematic review on the topic.

Recently, literature has been consistently raising concerns regarding the influence of spine deformation and its influence on shoulder mobility as well as the joint function after total shoulder arthroplasty [7, 23, 24].

Barret et al. showed how Thoracolumbar flexion (coef = 0.670) and lumbar lordosis (coef = 0.414) regions of the spine might be potentially overlooked when completing shoulder ROM assessment in asymptomatic individuals as more emphasis is placed on thoracic contributions to shoulder function [3]. Evaluating patients in a slouching position has shown a reduction in global shoulder ROM for some authors [4, 17], while only in abduction for others [14]. The relationship of this angle with shoulder ROM and clinical outcomes can be attributed to several factors. First, the scapula protracts and downwardly rotates when bending forward, leading to compression under the acromion of subacromial tissues. Second, since extension of the thoracic spine should be required to elevate the shoulder, complete elevation of the shoulder with an increased kyphosis cannot be achieved.

Moreover, forward scapular posture influences altered scapulothoracic muscle imbalance and scapular kinematics, which could lead to shoulder impingement syndrome and rotator cuff disease [19, 22].

Muscle strength and length are also factors related to scapular kinematics and shoulder ROM, with particular focus on the strength of the serratus anterior and the length of the pectoralis major influencing anterior tilting and scapular protraction [5]. The results of the analysed data in present study support this theory [16]. Also, the serratus anterior and the lower trapezius influence the scapular movements, stabilising the inferior angle and the medial border of the scapula, directly protecting form anterior tilt [8, 20].

Even if spine and scapular imbalance have been shown increased in the middle‐aged and elderly people [11, 12], recent findings show that an increase in scapular internal rotation is attributed to posture rather than age, highlighting how muscle strength is essential to maintain the spine sagittal alignment and the physiological scapular orientation [23].

The relation between spine and shoulder function has been investigated in the shoulder arthroplasty surgical setting, where different postures influenced the 2‐year clinical outcome of patients in terms reducing flexion, abduction and exacerbating pain after surgery [24].

These findings led the orthopaedic surgeons to consider the correct orientation of the prosthesis in relation to the scapula, spine and humerus morphology to achieve the best clinical scores and the highest patient′s satisfaction [1, 23, 24, 29].

Anatomic total shoulder arthroplasty and reverse shoulder arthroplasty are effective treatment options for degenerative diseases of the glenohumeral joint, however, appropriate positioning of the components is among the most relevant factors to succeed in this type of surgical treatment [9, 10].

The relationship explored in this study between spinal morphology and shoulder function resembles the well‐documented pathogenesis of hip‐spine syndrome, characterised by lumbar degenerative stenosis and hip Osteoarthritis [25]. As alterations in the alignment and function of the lumbar spine can impact hip mechanics and vice versa, the findings of this review suggest a similar relationship between the dorsal spine and shoulder. In both cases, biomechanical correlations imply that altered function or alignment of one segment can have a detrimental effect on the ROM and overall function of the associated joint. This similarity demonstrates the need for a holistic approach to musculoskeletal assessments: similarly to the hip‐spine syndrome, effective treatments require diagnosis and management of the primary aetiology and associated secondary effects [27, 30]. This systematic review has some limitations. First, in the current literature, there is a low number of high‐level studies. Also, the use of heterogeneous ROM measurement and functional scores, different sample sizes, and a wide age range might bias or obscure the observed correlations. Patient‐reported outcomes measures (PROMs) were not considered or analysed in relation the ceiling effects (CE). CE may arise when the maximum achievable score on a given PROM is attained by at least 15% of the study population. The presence of a CE typically suggests that the scoring thresholds are insufficiently challenging, potentially limiting the measure′s ability to differentiate higher levels of patient function.

Moreover, the high heterogeneity of the included studies, the functional scores used and the evaluated outcomes did not allow for a meta‐analysis. Finally, other limitations are related to the small sample size and the different short‐term follow‐ups. A specific analysis on how forward scapular posture interact with thoracic kyphosis to influence shoulder kinematics should be conducted to gain more personalised insights into the treatment of shoulder pathological conditions.

## CONCLUSION

This study agrees with the hypothesis and underlies a frequent significant correlation between spine morphology, shoulder function and scapular orientation, especially between shoulder abduction, adduction and flexion with thoracic kyphosis. These results should be considered in the clinical practice to treat and to better align patients and physicians on expectations after a surgical or a non‐surgical shoulder treatment. Further research and higher quality studies should be carried out to individuate the better define these frequent correlations and their applications in the daily clinical practice.

## AUTHOR CONTRIBUTIONS

*Conceptualisation*: Pietro Gregori and Fabrizio Russo. *Methodology*: Mauro La Bruna and Giuseppe Francesco Papalia. *Software*: Michele Paciotti. *Validation*: Giancarlo Giurazza, Mauro La Bruna and Clemente Caria. *Formal analysis*: Clemente Caria. *Investigation*: Mauro La Bruna. *Resources*: Rocco Papalia. *Data curation*: Michele Paciotti. *Writing original draft preparation*: Pietro Gregori and Giuseppe Francesco Papalia. *Writing review and editing:* Rocco Papalia. *Visualisation*: Umile Giuseppe Longo. *Supervision*: Rocco Papalia. *Project administration*: Edoardo Franceschetti. *Funding acquisition*: Edoardo Franceschetti. All authors have read and agreed to the published version of the manuscript.

## CONFLICT OF INTEREST STATEMENT

The authors declare no conflicts of interest.

## ETHICS STATEMENT

None declared.

## Data Availability

All data generated or analysed during this study are included in this published article [and its supplementary information files].
